# Supplementary material for: Hospital-based evidence on cost-effectiveness of brucellosis diagnostic tests and treatment in Kenyan hospitals
Source: PLoS Negl Trop Dis. 2021 Jan 7;15(1):e0008977. doi: 10.1371/journal.pntd.0008977 (PMC7833147; doi:10.1371/journal.pntd.0008977)
Supplement: Attachment — Submitted filename: Reviewer response.docx [file pntd.0008977.s001.docx]

**Editor response:**

*Dear Editors,*

*Many thanks for the opportunity to edit our manuscript in light of the reviewers’ comments. We found the comments very helpful, and their implementation has greatly improved our manuscript. We are therefore very thankful for their time.*

*We are also grateful to the journal editors for granting us an extension to the submission deadline.*

*Below please find our detailed response to each comment. For the sake of brevity we have not included the modified sections within this document, but we have included manuscript line numbers and all changes are highlighted in the revised manuscript.*

*Kind regards,*

*Laura Falzon*

**Reviewer response:**

**METHODS:**

**Reviewer #1:** Authors use data collected from hospitals in Kenya and results obtained with the same patient sera in both tests to calculate their cost-effectiveness, from a societal perspective, defined as the cost in US Dollars ($) per Disability Adjusted Life Year (DALY) averted over the course of one year. The objective of the work is clear and the design of the study, sample size and statistical analysis seem appropriate to address the stated objectives. According to the manuscript, ethical and regulatory requirements were met.

However, there are several gaps and inconsistencies in the manuscript that need to be clarified and properly described before considering the work for publication:

1. The major gap of the article is the lack of a precise description of the sera used for comparison. In the abstract it is stated that “serum samples that were tested for brucellosis using the FBAT, were later re-tested using the RBT”. It is also indicated: “FBAT results were available for 205 patients, of which 30 (15%) tested positive”. Accordingly, these 205 serum samples should have been re-tested with the RBT. Surprisingly, it is indicated (abstract and lines 263-264): “A total of 255 serum samples were collected from the 10 hospitals, and these were re-tested with the RBT”. Thus, results in Table 2 are confusing since the number of sera tested in each test is different and the corresponding number of positive/negative is not indicated.

*We apologize for the confusion in how we reported the number of samples tested. We have now adjusted the numbers so as to report results only for those samples that were tested with both diagnostic tests (n=180).*

*These adjustments were made in the Abstract [lines 23-25], Results [lines 276-286] and in the revised Table 2.*

In addition to an error in the percentage of FBAT positive samples in hospital A (should be 50% instead of 5%),

*Thank you for highlighting this error. We have corrected this typo in Table 2.*

Other source of confusion comes from data in hospital J since it is impossible to assess if the only RBT positive sample identified was previously positive or negative in the FBAT, and if the 2 FBAT positive samples were negative in the RBT. The correspondence of FBAT/RBT results cannot be assessed also in the other hospitals.

*To address this comment, we have added a couple of sentences in the Results section to clarify which of those samples that tested positive with RBT, were also positive with FBAT [lines 283-286].*

If the objective is to show the correspondence among FBAT and RBT results this Table should be fully amended indicating clearly which FBAT positive/negative sera were also positive/negative in RBT. However, authors should consider if this table could be unnecessary and redundant with data in Table 3.

*Table 2 has been amended to only include results for the 180 samples for which both test results were available, while Table 3 has been removed since it was deemed redundant (and based on another reviewer’s comment to reduce the number of Tables). The raw data will be uploaded to an open access repository which will be referenced in a final paper. In the meantime, the raw data can be provided upon request should the reviewer wish to see them prior to uploading.*

To increase the mess, authors indicate (lines 265-266): “Both FBAT and RBT results were available for 180 samples (Table 3)”, and the relative DSe/DSp and relative (to be amended along the manuscript) False Positive/Negative values calculated.

Altogether, the precise number of sera tested should be well defined in the material and methods, and the corresponding tables amended/merged.

*Once again we apologize for the confusion regarding the sera samples. As mentioned above, we have now amended all relevant sections (abstract, results and Table 2) to only include results for those sera which were tested with both tests (n=180).*

*We have also decided to omit the section on DSe/DSp and False Positive/Negative values to avoid confusion.*

In addition to the precise description of the sera used, the material and methods section should be properly completed with a description of the FBAT test (origin, antigen composition, method, etc…). To have a better (but yet incomplete) idea on FBAT methodology is necessary to read lines 289-293 (i.e. the results section).

*Details on the FBAT kits used in the participating hospitals have been added in Table 2 and in the Results section [lines 300-302 and 310-322].*

Moreover, comments in lines 256-261 should be explained better. It is stated that four hospitals conducted FBAT with both A and M antigens. Which FBAT antigen (A?, M?) was used in the remaining hospitals? It would be probably more practical avoiding all these comments (based on inadequately assumed test properties) in the results section and make suitable criticisms of FBAT in the discussion section (that in fact were made yet -lines 346/347-).

*As suggested by the reviewer, we have added details on the FBAT kits and antigens used by the participating hospitals in Table 2. We have also removed the section that described the separate antigen results, and only maintained a brief criticism of this in the discussion section [lines 392-394].*

It is indicated that Scenario 2 was built according “Brucella spp. prevalence in febrile patients presenting to hospital in North-East Kenya based on real time PCR”. This seems to be out of the context of the study. If this test is to be included in the comparative study, a precise description of the PCR protocol should be made in the material and methods section, and moreover, the correspondence with the serological results properly described also. Otherwise this scenario should be omitted from the manuscript.

*The aim of Scenario 2 is to illustrate the influence of brucellosis prevalence on the cost-effectiveness of a change from FBAT to RBT. The underlying assumption here was that in the Northern Region the prevalence of 15.4% by PCR was a ‘true’ prevalence, and we did not wish to compare the use of the FBAT or RBT with PCR. Rather, the model then used the assumed Se/Sp of FBAT and RBT to determine the CEA of the use of these tests in a high prevalence area. We could of course just illustrate this through a sensitivity analysis of prevalence but wished to illustrate our findings based on a real-world situation. We have clarified that this was used as an illustration in lines 207-211.*

**Reviewer #2:** The study design is appropriate to address the stated objectives of seeking hospital-based evidence on brucellosis diagnosis.

*Thank you.*

**Reviewer #3:** The statement of aims and objectives could be clearer. I’d suggest replacing the final introduction paragraph which covers the background to the study with a clearer statement of study objectives.

*We have revised the final introduction paragraph to include a clearer statement of the study objectives [lines 112-119].*

Can you clarify if you collect any individual level data on the actual treatments prescribed for the individuals whose samples were tested? This is not really clear from the methods.

*We apologize that this was not very clear. All data were collected at the hospital-level, and we have included a statement to clarify this in the methods section [lines 151-152].*

If the only data gathered were the ‘recommended’ treatment regimen for brucellosis at hospital level then more discussion on the limitations of this approach is needed. The recommended treatment regimen is likely to be a poor predictor of actual practice which will vary by clinician and all clinicians are likely to put < 100% FBAT positive individuals on brucellosis treatment. Your estimates of costs due to FBAT will therefore be inflated.

*We thank the reviewer for raising this issue. We went back to the field to inquire about this, and the participating clinical officers confirmed that they prescribe treatment to all patients that test positive with FBAT. However, they could not say how many of those patients would then go on to purchase the treatment as this would vary depending on the individual’s capacity of paying and/or availability of health insurance. In the absence of more precise knowledge on patient’s compliance, and how this may change when using different tests, we felt it was more appropriate to assume that all positive individuals get fully treated. However, we acknowledge the effect this assumption may have on the estimates of the cost-effectiveness analysis, and have discussed it as a limitation in the Methods [lines 238-242] and Discussion sections [lines 479-491].*

As you compare the FBAT to the RBT – some content in the introduction on the known limitations of the RBT would be helpful to prime the reader to better interpret the data presented. The implications for the interpretations of the study findings are not large – but should be made clear throughout. Content on this is given in the discussion but could be moved to the introduction instead.

*As suggested by the reviewer, we have included more information on the FBAT and RBT in the introduction [lines 97-104].*

Can you clarify the ‘inclusion criteria’? In a few places you talk about the implications of these findings for inferences about febrile populations but I think these data are specific to individuals who are tested for brucellosis based on clinical judgement? While these populations are likely to overlap not all individuals tested for brucellosis will be febrile and <100% febrile presentations are likely to be tested for brucellosis?

*We have included a statement to clarify when clinical officers would usually test a patient for brucellosis [lines 135-137]. We have also amended the sentences to specify that the data are specific to febrile patients who are tested for brucellosis based on clinical judgement [lines 256-257 and 495-496].*

There is no mention of power. Given the relatively limited size of the sample set, small number of positive individuals and wide CI on many estimates this should be explicitly acknowledged.

*Given the nature of the study, there was no estimated sample size and corresponding power. We have however acknowledged the limited size of the study in the Discussion [lines 500-502].*

In specifying the betapert parameters can you clarify the rationale for the selection of the max and min values specified? It looks like you have used the 95CI values (e.g. for p4) which perhaps sets the distribution quite conservatively narrow (e.g. using 5% and 95% as 0 and 100% values)? Is this standard?

*Thank you for this useful comment which we have taken on board. Indeed, the use of the 95% CI to parameterise a BetaPERT distribution does appear to be inappropriate. We have therefore used the AusVet tool https://epitools.ausvet.com.au/ to estimate Beta distributions from count data where appropriate and used the distribution fitting function in @Risk to parameterise our raw data (cost of diagnostic test and treatment) or a uniform distribution where only a range was available. The distributions were then truncated using RiskTruncate(0,1). The new method is described in lines 190-204. You will observe that the 95% confidence intervals around our cost-effectiveness estimates have widened correspondingly.*

Also – please provide more information on which FBAT the literature estimates used to parameterise this model are based on (from Kiambi et al study) and how this relates to the tests used to generate the data in this study?

*We have included information on the FBAT used to parameterise this model in the methods section [lines 194-198], and how this relates to the tests used to generate the data in this study in the discussion section [lines 419-422].*

Data that I am aware of (admittedly unpublished) would indicate that you’d need a much wider CI on this value to capture e.g. the range of performance of 4 FBAT assays (~ 10-80%) so I would be interested in the implications of a less precisely specified parameter for the findings of this analysis.

*The sensitivity of the FBAT has a large impact on the outcome ($/DALY averted) as indicated in the graph below from a sensitivity analysis of different values of Se from 10-80%. However, without more information (published) on the performance we have opted to stick to the data we have identified on the performance.*

Mean of $/DALY averted -FBAT -Busia B50 vs Percentage Change of Inputs

$/DALY averted -FBAT -Busia

| Change From Base Value (%) | P(true positive FBAT) / Western Kenya B5 |
| --- | --- |
| -72.973% | 8380.92 |
| -45.9459% | 4200.26 |
| -18.9189% | 2806.71 |
| 8.10811% | 2109.93 |
| 35.1351% | 1691.87 |
| 62.1622% | 1413.16 |
| 89.1892% | 1214.08 |
| 116.216% | 1064.77 |

P13 is based on very limited data I believe (n=2 observations. Allowing a little more variation in this parameter would seem sensible – not least because, as you indicate costs might well be lower at scale.

*This is a very reasonable point. Given the paucity of data for this parameter we have extended our uniform distribution from $1.5 – $3.26 (Table 2). Within a larger study based in the same study area (Falzon et al, 2019), we calculated a cost/sample of $3.26 per sample for collection and running the test, including all consumables and technician time. We have therefore set this as the upper estimate of costs (being that we were purchasing on a relatively small scale compared to a national health system and used an expensive blood collection system), costs below $1.5 are unlikely if all consumables and staff time are considered. We have included a statement on this in the results section [lines 324-332].*

P15 – how do you specify a 3 parameter distribution with the 2 values given?

*Thank you for pointing this out. Looking back at the data provided we feel it is more appropriate to present this parameter as a uniform distribution between the stated minimum and likely maximum DALY burden (0.15-0.211) (lines 203-204 and Table 1).*

Line 233-234: can you add more detail on the methodology applied for a ‘basic sensitivity analysis’?

*We have added a short paragraph on this to the methods section [lines 260-264].*

It is important that you include more details on the actual FBAT kits used – e.g. manufacturer and product names. There are a variety of options in the setting I am familiar with and their performance is quite variable. I think it is important to make this clear and move away from the impression that you give that the FBAT is a single fixed test.

*Thank you for highlighting this aspect. We have included information on the manufacturer and product names of the FBAT kits used in the hospitals in the revised Table 2. We have also included more information on these test kits in the results [lines 310-322] and discussion [lines 413-416] sections.*

In Table 1 – can you clarify which FBAT the estimates for Parameters 4-7 were estimated using? Are these appropriate for all of the tests used for this study?

*We have included information on the FBAT used to parameterise this model in the methods section [lines 194-198], and how this relates to the tests used to generate the data in this study in the results [lines 315-317] and discussion [lines 420-422] section.*

I appreciate the importance of the comparison of the relative performance of the FBAT and RBT but the absence of any additional confirmatory testing of these samples is a limitation that should be recognised.

*We agree with the reviewer; for the sake of clarity we have omitted the section describing the relative sensitivity and specificity of the two tests, and have limited our comparison of the two tests to the Kappa agreement. We also mention this as a study limitation [lines 502-503].*

It is also important that you clarify the need (according to all of the guidelines that I am aware of) for a confirmatory serological test. Confirmatory testing may not be performed in practice for many individuals but should be mentioned before the section in the discussion which indicate that secondary tests are as an option.

*Thank you for this clarification. We have amended the sections related to this in the author summary [lines 39-41] and in the discussion [lines 514-515].*

In the methods (line 137) you refer to extracting data on patient ID but there is apparently no consent obtained from participants. Why not? Please give some additional explanation of the handling of personal identifying info and why consent was not obtained.

*Our ethical permission (IREC Reference No. 2017-08)* *allowed us to make secondary use of data that did not involve patients directly. In this study we made secondary use of anonymised hospital samples, and did not handle any personal data. All serum samples were labelled with a unique number which was not linked to the patient in any way. We recognize that the term “patient ID” was confusing and have therefore replaced it with “unique identifier” [lines 132 and 138-139].*

**Reviewer #4:** Introduction: Please tighten the write up, keeping it crisp and relevant to the topic.

*We have revised the Introduction and omitted some paragraphs to keep it more succinct and to the point.*

L112-119: The quote from the worker is not necessary. Please remove.

*As suggested by the reviewer, we have removed the quote.*

Please make objective (s) of the study explicit and clearer. In my opinion, there are several objectives in this study. Hence, these need to be explicitly stated but at the same time, relevant to each other.

*We have revised the final introduction paragraph to include a clearer statement of the study objectives [lines 114-117].*

Materials and Methods: Was permission requested from the hospital management and administrator or Ministry of Health to conduct the study? If it was, please incorporate. The way it is written suggested that only the clinician or lab technologist were communicated.

*Indeed, following ethical clearance, ethical permission to conduct this study was obtained from the Ministry of Health and hospital management. We have clarified this in the ethical approval statement [lines 268-270].*

Please include ethical clearance ID (or other identification) from the board.

*This study was approved by the Institutional Research Ethics Committee (IREC Reference No. 2017-08) at the International Livestock Research Institute, and this statement has been included in the Ethical Approval section [lines 266-268].*

Please include description on why those hospitals were selected, it is not clear if these are all available hospitals in the study regions. Include criteria for selection if any.

*We have included a statement to clarify why the hospitals were selected, and their representativeness, in the methods section [lines 124-129].*

Please arrange and flow the materials and methods according to the objectives statement.

*As suggested by the reviewer, we have revised the materials and methods section so the order of the methods better corresponds with the listed objectives.*

Please make study design and sample size determination clearer.

*In this manuscript we describe a sequence of events, and we therefore did not follow a specific study design, not did we have a pre-determined sample size. We have tried to adjust the flow of the methods to better match the objectives and make this clearer. We have also included a statement acknowledging the limited power of the study in the discussion [lines 500-502].*

It is not clear what Levels of the hospital mean, is this relevant to the design of the study? Please clarify or remove.

*We apologize for the lack of clarity regarding these terms and have chosen to remove them from the text.*

Please be clear about the published Se and Sp of each test. Since the inaccuracy of FBAT was noted several times by the author and forms the backbone of the study, the Se and Sp should be made explicit. It would also be useful to note how the cited authors determined that FBAT diagnostic ability is poor.

*The statement on the poor diagnostic ability of the FBAT is based on our field experience and on previously published literature, as mentioned in the introduction section [lines 97-100].*

*The sensitivity and specificity parameters for the FBAT and RBT used in the cost-effectiveness analysis are also based on previously published literature, and we have clarified this in the methods section [lines 194-198] and in the revised Table 1.*

*To avoid confusion, we have removed the section in which we described the relative sensitivity and specificity of the FBAT based on our study data as we recognized the limitations of this approach.*

In my understanding, the authors attempted to determine the diagnostic accuracy of FBAT by comparing its performance to RBPT which is not a gold standard reference. Although the Se and Sp of the two imperfect tests were not stated, the authors stated that RBPT is the more accurate test therefore used the findings from the RBPT test to calculate the ‘relative’ Se and Sp of FBAT. At the same time the agreement was also calculated. Since the Se and Sp were not stated and there was no attempt by the authors to determine the true positive and true negative of RBPT before comparison to FBAT was performed, there could be questions about misclassification bias when RBPT diagnosis was then used to compare to FBAT and these were then used as input for the mathematical models.

*We thank the reviewer for highlighting this aspect and apologize for the confusion. As mentioned above, the sensitivity and specificity parameters for the FBAT and RBT used in the cost-effectiveness analysis are based on previously published literature, and we have clarified this in the methods section [lines 194-198] and in the revised Table 1.*

*Furthermore, we acknowledge that the computation of the relative sensitivity and specificity of the FBAT based on our study data were inappropriate given the lack of a confirmatory test, and we have therefore omitted this section.*

Please also look into using the method suggested by Albert (Estimating diagnostic accuracy of multiple binary tests with an imperfect reference standard. Stat Med, 2009) to determine the diagnostic accuracy of FBAT.

*We thank the reviewer for this suggestion. However, we lack the statistical power to formally undertake comparison of diagnostic tests as this was not the main focus of the paper. As mentioned above, we have chosen to omit the section on the relative sensitivity and specificity of the two diagnostic tests.*

Please be clear on what is meant by ‘societal perspective’ for cost effectiveness.

*We have edited the sentence to clarify the concept [lines 186-187].*

L185 – L193 is very confusing, please rectify.

*This paragraph has been re-written [lines 205-217].*

L244 – records cannot be tested for brucellosis. Please revise.

*We have revised this sentence accordingly [lines 278-281].*

Please revise comparison of the two tests considering my above comments on Se and Sp.

*As mentioned above, we have omitted the section describing the relative sensitivity and specificity of the FBAT.*

It was not clear if both RBPT and FBAT were performed by the authors or if only RBPT was performed and results were compared to FBAT results performed by the hospitals.

*The FBAT was performed by the hospitals, while the RBT was performed by the authors at our field laboratory in Busia. We have revised the corresponding sentences in the abstract [lines 23-24], methods [lines 172-173], results [lines 278-279 and 282], and discussion [lines 395-396] sections.*

**RESULTS:**

**Reviewer #1:** Main concerns on results and discussion are: Table 4. It is remarkable (and incomprehensible to this reviewer) why the same treatment is 5 times more expensive in hospital H than in hospital B. If this is not an error, this finding should be better commented and discussed properly.

*We also found the discrepancy perplexing. However, we confirmed with the pharmacists at both hospitals that these costs are correct. They explained that the likely reason for the discrepancy is that Hospital B is a government funded public health facility where costs are often subsidized to promote equity in access to services and individual treatment. Hospital F, on the other hand, is a private facility where costs are higher since patients can afford health insurance or out-of-pocket payment. We have modified Table 3 to include details on the type of hospital, and we have included a statement to explain this discrepancy in cost treatment in the discussion [lines 425-430].*

Lines 357-358: that the “prozone” is the only (or the major) responsible of the poor diagnostic performance of FBAT is very questionable. Improper setting up (antigen composition and preparation) and validation, among other problems, can explain also the poor FBAT performance.

*We thank the reviewer for highlighting this. Based on other comments to tighten the write-up and avoid speculation on the FBAT results, we have omitted this section in the discussion.*

Lines 463-465. The consideration regarding the suboptimal specificity of the RBT is not correct and I suggest a careful revision of this sentence. The great sensitivity of RBT to detect Brucella specific antigenic stimulus in endemic settings can never be considered as a lack of specificity.

*We agree with the reviewer on this and apologize for the confusion. Based on other comments to tighten the write-up and improve the flow of the discussion section, we have omitted this section in the discussion.*

Lines 475-476: In brucellosis, the concept of “screening” and “confirmatory” tests is ambiguous and frequently misinterpreted. In animal brucellosis (when vaccination is used), a serial RBT (as “screening”)/CFT (as “confirmatory”) testing can be of value to minimize the number of unnecessarily culled animals. However, in human brucellosis the RBT is not a “screening” test that, in opposition to other diagnostic tests, needs to be “confirmed”. If properly performed the RBT is specific enough and results in the same diagnostic performance as, for example, SAT, ELISA and LFIC. Importantly, the results of any indirect diagnostic test have to be contrasted by the clinician with the epidemiological and clinical evidence and, if available, cultures. I strongly suggest authors to discuss this issue with enough care to avoid perpetuate this misconception.

*We also agree with the reviewer on this. As mentioned above, however, we have omitted this section in the discussion to tighten the write-up and improve the flow of the discussion section.*

**Reviewer #2:** The results are clearly and completed presented. The two legends in Figures 4 and 5, Input High and Input Low, are difficult for the readers to distinguish.

*Thank you for pointing this out. We have revised the two Figures (now Figures 3 and 4).*

**Reviewer #3:** Can you give the raw data on test results so that the breakdown by FBAT manufacturer and hospital is clear.

*Table 2 has been amended to include the type and manufacturer of the FBAT kit used at each hospital. The raw data will be uploaded to an open access repository which will be referenced in a final paper. In the meantime, the raw data can be provided upon request should the reviewer wish to see it prior to uploading.*

You have the hospital breakdown in Table 2 – was the same test used for all tests at a given hospital?

*Different hospitals used different FBAT kits. As mentioned above, in Table 2 we have included the type and manufacturer of the FBAT kit used at each hospital.*

Can you add the detail on the tests used? The FBAT is not a single test so this needs to be clear in the presentation of the data and in the discussion of the results.

*Details on the FBAT kits and antigens used in the different hospitals have been added to Table 2 and in the results [lines 300-302 and 310-322] and discussion [lines 413-416] sections.*

You state a series of assumptions (pg 11) but don’t really come back to many of these in the discussion. How plausible is to that each patient treated undergoes a full brucellosis treatment protocol? Or that patients seek care at the onset of symptoms? Given widespread awareness of the limitations of brucellosis testing (amongst patients and clinicians) might assumption 7 not change with improved diagnostics? What are the implications of violating these assumptions for the interpretation of your findings?

*We agree with the reviewer that the assumptions will likely influence our cost-effectiveness estimates. However, given the paucity of data on patient compliance, and how this may change with improved diagnosis, we do not feel comfortable modifying these assumptions. Nonetheless, we have included some text in the Discussion to acknowledge these limitations [lines 475-491].*

You recognise the assumption that all individuals who get a positive test result are treated for brucellosis. In my experience only a fraction of individuals receiving an FBAT positive result are put on brucellosis consistent treatment regimens. As you recognise in the paper this is largely because clinicians are well aware of the limitations of these tests. Can you include a parameter for % test positive individuals treated for brucellosis? I’d imagine this will vary with both prevalence and PPV of the test used.

*As mentioned above, we went back to the field to inquire about this. The participating clinical officers confirmed that they prescribe treatment to all patients that test positive with FBAT [lines 333-334]. However, they could not say how many of those patients would then go on to purchase the treatment as this would vary depending on the individual’s capacity of paying and/or availability of health insurance. In the absence of more precise knowledge, and since the paper was not about compliance but rather on the cost-effectiveness of the treatment given the assumption that the national guidelines are adhered to, we felt it was more appropriate to assume that all positive individuals get fully treated. However, we acknowledge the effect this assumption may have on the estimates of the cost-effectiveness analysis, and have discussed it as a limitation in the Methods [lines 238-242] and Discussion sections [lines 479-491].*

Line 244-and table 2: As the bulk of your analysis depends on data where both the FBAT and RBT were performed (n=180), the data presented in table 2 could be made much clearer. If “Serum samples available” = “n tested by RBT” can you make this clearer. Can you give clearer labels to the 2 columns currently headed “% positive” to differentiate the tests.

*We apologize for the confusion in how we reported the number of samples tested. We have now adjusted the numbers so as to report only on those samples that were tested with both diagnostic tests (n=180).*

*These adjustments were made in the Abstract [lines 23-25], Results [lines 276-286] and in the revised Table 2. We have also modified the labels of the two columns in Table 2.*

Can you also clarify which if any estimates presented or used in the model use a different denominator – ideally stating the population clearly in each case.

*Parameters on diagnostic test performance used in the mathematical model were all derived from published literature, as mentioned the methods section [lines 190-198] and in Table 1. The only parameters that were based on data from this study were P1 [the number of patients tested per year], P12 [cost of FBAT to patient], P13 [cost of RBT to patient], and P14 [cost of antibiotic treatment].*

*To avoid further confusion, we have omitted the section on the relative sensitivity and specificity of the FBAT based on our study data.*

Lines 256-261 – can you give a more systematic presentation of these data (and the methods followed to generate them)? Who did you ask about test info? What were the manufacturers of the tests? Which hospital was each test used in? What data do you have to support the statement that ‘consequently a prescription was provided by clinicians’. Did you ask explicitly if all test positive individuals were put on brucellosis treatments? How plausible do you think that is?

*We have added more information on this in the methods [lines 143-152] and results [lines 309-322 and 333-340]. We have also included information on the test kits used in each participating hospital in the revised Table 2.*

Much of the data on test results for abortus and melitensis antigens is not key and presenting them risks giving these tests a validity that is not warranted. Rather than presenting these results here you could instead include a clearer statement of the definitions of ‘test’ positive by FBAT that should be given in the methods. E.g. for FBAT, individual classified as positive if any reaction at any dilution with any of the antigens in the specific kit? The details of the actual kits used are needed here and any information gathered on the way they are applied. Also – presumably FBAT positive means reported positive by any protocol reported – with or without dilutions etc? This is important detail to give to enable your reader to determine the validity of comparing the FBAT and RBT, and handling of the FBAT as a single test.

*As suggested by the reviewer, we have removed the section describing the test results for abortus and melitensis antigens, and have instead included a statement on when a test was considered positive in the methods section [lines 133-135]. We have also included information on the actual kits and how the test was carried out in the participating hospitals in Table 2 and the results section [lines 310-320], respectively.*

Line 263 – value of RBT testing sera for which no FBAT data are available is not clear? Why was this done?

*We agree with the reviewer and apologize for the confusion created in this regard. We have now adjusted the numbers in the Results section [lines 276-286] and in Table 2 so as to report only on those samples that were tested with both diagnostic tests (n=180).*

There are results presented in this section that aren’t really introduced in the methodology. Some more detail on the respondents per hospital and the exact questions asked to obtain the data presented would be helpful.

*We have added more information on the respondents per hospital and the questions asked in the methods section [lines 143-152].*

Lines 288-294 – You have mentioned the protocol options given in test kit inserts but not the details of the protocols actually run at the hospitals. Did any run dilutions - slide/ or tube format? If so, did you specify a common protocol for the question about time to perform one test?

*We have included information on how the FBAT was carried out at the participating hospitals in the results section [lines 315-320].*

You mention the publication dates of the references in the test protocols but make no point about this in the discussion – presumably the implication is that these are outdated kits/technology?

*We have included a statement on this in the discussion [lines 417-418].*

Line 294 Can you clarify the question asked to obtain the ‘mean cost of a test/patient’ in the methods and indicate clearly if this is a cost per test or per patient here.

*We have attempted to clarify this in the methods [lines 179-181] and results [lines 321-322] sections.*

Line 300 – given that treatment regimens are either consistent with guidelines or not and that durations will vary by drug I am not sure how meaningful a mean treatment duration is?

*We agree with reviewer and have edited this sentence accordingly [lines 335-336].*

Line 308 – language point – I think the shift to RBT would be cost-effective or not as compared to the FBAT? Vs more cost-effective.

*We have revised this sentence accordingly [lines 346-348].*

Lines 325-328 – it would be helpful to spell out the directions of the relationships for the most influential parameters – e.g. lower prevalence > what implications for cost differences? In what contexts would cost differential be most pronounced? You mention this in the discussion (lines 458-460) but it takes a long time to get to.

*Thanks for this remark, we have given some basic explanation of the direction of the relationships in the results section [lines 367-371].*

Table 2 – Can you include the kit name/manufacturer info for the FBATs used.

*As suggested, in Table 2 we have included the name and manufacture of the FBAT kit used in each hospital.*

Table 4 – Please give more detail in the methods on the questions asked about the “protocol of the FBAT”. Was this “how long does it typically take to run the test?” or other details also? Is the ‘test cost’ the charge to the patient or another quantity?

*We have added more information on the respondents per hospital and the questions asked in the methods section [lines 143-152].*

Please include rows for all hospitals with NAs indicating missing data as needed?

*As suggested by the reviewer, in Table 4 we have included rows for all hospitals with NAs to indicate missing data.*

Can you add a column to indicate which of these treatments regimens are consistent with WHO guidelines?

*As suggested by the reviewer, in Table 4 we have added a column to indicate which of the treatment regimens are consistent with WHO guidelines.*

Figures – can you plot the distributions of the $/DALY averted estimates for each scenario – not just for scenario 1 where prevalence is very low and include the plots on a single figure to aid comparison (e.g. table 5 as graphic)

*Overlaying all distributions was not very easy to interpret. We have therefore opted to have a series of three figures (Figures 2a, b and c) which demonstrate the relative frequency of each $/DALY averted result in the simulations for the two tests under the three scenarios*

Can you include more detail in the legends for figures 4 and 5 – e.g. what is “DW”? And other abbreviations used.

*The figure legends have been addressed and abbreviations avoided.*

In the discussion of the $/year for treatment of false positives (re results in Table 5 and Lines 318-324) it would be useful for you to compare the costs for brucellosis treatment to the costs for treatment for other causes of febrile illness. Given that the patients currently testing positive by FBAT would be treated for something if not brucellosis and that this would presumably have some cost also are the values for $/year treating false positives not overestimates?

*We appreciate that the treatment of febrile illness in such an environment is subject to many issues, and that some diseases may overlap. We also agree that the investigation of the ancillary benefits of over-treatment merits future research. However, since this study was focusing on brucellosis, and since discussion of this would require data on other febrile diseases in this study area and a whole set of assumptions which go beyond the scope of the paper, we have opted to not delve into this any further within this manuscript.*

**Reviewer #4:** Please align results to the objectives and materials and methods.

*We have revised the manuscript to align the results with the objectives and materials and methods.*

Please improve how results are presented but keeping them concise and structured. At present, the results are hard to follow and since the objectives are not clear, it is hard to engage the results to a stipulated objective.

*We have revised the results section, and aligned their presentation with the objectives.*

There are too many tables and figures in this manuscript, please consider merging some and deleting others that has been described in text. Some of the figures are hard to read, please improve quality.

*We have removed one of the Tables (what was previously Table 3), and we have revised the figures to make them clearer.*

**DISCUSSION & CONCLUSION:**

**Reviewer #1:** Conclusions of the study are right and provide a useful support to hospitals to address a public health relevant problem (brucellosis), particularly in scarce resource areas.

*Thank you.*

**Reviewer #3:** -Are the limitations of analysis clearly described? Not all – Some more explicit discussion of the fact that the estimates for cost of treatment based on FBAT are likely overestimates as not all individuals with positive test results will be put on brucellosis treatment is needed.

*We have included some text in the discussion to describe this limitation [lines 479-491 and 503-505].*

Lines 339-341 might be a bit premature. While this statement is hopefully true, ‘will’ might not be the best term. I also think it important to recognise the limited scale of this study in the discussion.

*We have revised this sentence accordingly [line 383], and have also included a statement on the limited scale of this study in the discussion [lines 500-502].*

**Reviewer #4:** Much of the discussion needs to be revisited after taking account the diagnostic accuracy of FBAT. Please improve conciseness, reduce wordiness and flow throughout.

*We have revised the discussion to improve the flow and reduce wordiness.*

L349-353 – this is speculation, unless there is type of suggestive evidence please remove.

*As suggested by the reviewer, we have removed this section.*

Include limitations of the study, including limitation of the test method used, mathematical modelling and data collections methods.

*We have included a paragraph on the study limitations in the discussion section [lines 500-506].*

Please tie conclusion to objectives of the study.

*We have revised the conclusion to better align it with the stated study objectives [lines 508-523].*

**MINOR MODIFICATIONS:**

**Reviewer #1:** Minor corrections:

Line 70: Authors should not use clinical/epidemiological adjectives such as “chronic” brucellosis to define the clinical course of the disease. From the scientific standpoint it would be probably better use terms like “short/long evolution”.

*Thank you for pointing this out; we have revised the sentences accordingly [lines 60 and 82].*

Lines 110-119. I strongly suggest deleting this personal (literal) opinion of the officer, and to be replaced with a more clear and suitable comment to illustrate the issue.

*We have removed the officer’s quote and replaced it with a clear statement of the study objectives and rationale [lines 112-119].*

Line 178. Delete “from a societal perspective” since it is already indicated in line 176

*This phrase has been deleted.*

Line 185. To avoid difficulties to readers, I suggest introducing “The different parameters and scenarios for the comparative cost-efficacy analysis are shown in Table 1” or a similar sentence.

*We have included such a statement [lines 205-206].*

Table 1. Unless this be a rule of the journal, the meaning of P (probability) is missing. It should be indicated in the text (line 180. “associated probability (P)”) and in Table 1 (as foot note).

*We have included a footnote below Table 1 to clarify the meaning of (P) [line 223].*

Line 202. Replace “patents” by “patients”

*Thank you for pointing out this typo; we have edited the word accordingly [line 226].*

Figure 1. The meaning of P, Se, Sp, Dx and Tx is missing (it should be indicated in the figure caption). The abbreviations Se and Sp should be included also in the text (in brackets) the first time these terms appear.

*Thank you for this observation. We have indicated the meanings of the abbreviations in the figure captions.*

Line 320. Replace “under an RBT” by “under a RBT”

*This sentence has been edited accordingly [line 359].*

**Reviewer #2:** Page 18, there is a typo for the line ‘Hospital A’ in Table 2. For FBAT, % positive should be 3/6=50%, not 5%.

*Thank you for pointing this out; we have corrected the typo in Table 2.*

**Reviewer #3:** Line 40 and 65 – a single test is never sufficient to make a confirmatory diagnosis of brucellosis either. The inclusion of two tests in international guidelines should be made clear here.

*Thank you for this clarification; we have edited the respective sentences accordingly [lines 39-41 and 64-65].*

Line 83 – ‘sensitivity/specificity balance’ is quite vague. Can you clarify which test performance metrics are dependent what epi conditions?

*This sentence has been removed in an attempt to shorten the text.*

Line 90-91 – you imply that many studies from markedly different settings have all shed light on some common characteristics – or indicate endemicity – but this is not very clear. Can you clarify?

*We have edited the sentence to make it more clear [lines 69-71].*

Line 101 – given that you have described brucellosis as endemic in Kenya is re-emergence the right term -or can you provide more precision on the contexts referred to?

*The section describing the re-emergence of brucellosis has been removed in an attempt to shorten the text.*

Lines 120-126 – the narrative on the origins of the study is not really key to this paper. Can you give more info on the scientific rationale that presumably motivated some of the interest of stakeholders?

*This paragraph has been revised accordingly [lines 112-119].*

Line 142 – does the ‘diagnostic kit protocol’ mean manufacturer info and specifications on test performance? Could be clearer.

*We have revised this text to make it more clear [lines 143-147].*

Lines 160 – the reference to paper based records is not very clear – what records are these?

*We have revised this sentence [line 167].*

Line 188 – how realistic is this assumption that all parameters apart from prevalence would remain the same? See suggestion above about including additional parameter for % FBAT positive individuals put on treatment, which might be expected to depend on PPV, which in turn depends on prevalence.

*Thank you for this comment, we agree that the PPV of the test (relating to underlying prevalence as well as test performance) may influence the trust in the test by both clinician and patient, and therefore influence compliance with treatment. We do not have sufficient data on which to parameterise this but agree it is a key gap and have suggested in the discussion some further work which would improve the work we have presented [lines 479-491].*

Line 354- The prevalence estimates and other test performance metrics reported here are somewhat misleading as they apply to different populations – can you report the % positive and other stats obtained through analysis of the population tested by both tests (e.g. ~13.3% vs 1.66 %? for prevalence)

*We have revised the manuscript to only present results for those serum samples for which both FBAT and RBT results were available. This sentence in the discussion has therefore been revised accordingly [lines 395-396].*

Line 358 – do you know that the FBAT kits are all pH neutral? What data is this based on? Can you include a reference?

*Based on another comment, this section in the discussion has been removed.*

Line 380 – really? if malaria is likely to be tested for alongside brucellosis how likely is it (given the availability of high quality rapid tests for malaria, relative severity of the diseases and emphasis on malaria in clinical training as well as societal pressures not to miss malaria cases) that any/many cases of malaria would be missed and treated as brucellosis?

*We thank the reviewer for pointing this out, and agree that malaria is not likely to be missed as, in this study area, it is often the first disease to be tested for in a febrile patient. In an attempt to shorten the discussion, this section has been omitted.*

Lines 383 -the points about common risk factors for multiple zoonoses are not very clear here. Can you explain further?

*This section on other zoonotic diseases has also been removed to shorten the text.*

Line 400 – can you add the data on compliance with WHO recommendations to Table 4 vs reporting results in the discussion?

*As suggested by the reviewer, we have added a column in Table 4 to indicate whether the prescribed treatments complied with the WHO recommendations, or not.*

Line 409 – can you clarify what practice you are defining as ‘improper’ use of drugs in this paragraph?

*We have replaced the word ‘improper’ with ‘inappropriate’ [line 435].*

Lines 421 – the points you make here are entirely consistent with a scenario in which clinicians interpret the positive FBAT alongside their knowledge of the poor performance and low positive predictive value of these tests and other data and choose to put patients on a short course of antibiotics to try to treat multiple possible bacterial infections. In contexts that I have worked in clinicians given an initial treatment like short course of doxycycline and then only consider shifting to a brucellosis drug regimen if patients fail to respond to that treatment. What are the implications of such practices for your assumptions re treatment of all FBAT positives for brucellosis?

*We acknowledge that different clinicians are likely to prescribe different drugs for different reasons. We contacted the clinical officers who participated in our study, and they confirmed with us that they always prescribe a treatment when a patient tests positive, as indicated in lines 333-334. We realize that the patient compliance may then vary considerably, and have included text in the discussion to address this limitation [lines 479-491].*

Line 430 – what ‘other febrile issues’ do you mean?

*We have added ‘trypanosomiasis’ to clarify this statement [line 408].*

Line 434 – can you present the multiplier given here (9.2) explicitly in the results - with CI also?

*We have removed this comparator as we feel the figures stand alone.*

Line 439 – can you give a date for the $197 purchasing power parity figure – the language here is a little unclear on the comparability of these figures.

*We agree this was unclear and hope the revised text [lines 470-474] is clearer. We have used 2016 data as presented by IHME at 2018 purchasing power parity.*

Line 443 – I am confident that the RBT is cost effective vs the FBAT but if all FBAT positive individuals were put on brucellosis treatments that would likely be effective treatment of most bacterial infections that might have caused their illness – leading to relatively low rates of treatment failure and repeat visits perhaps? However, you have likely over-estimated the costs of the FBAT with this assumption so a bit more reflection on these assumption would be sensible.

*We have added text to discuss the limitations of this assumption in the discussion [lines 479-491] and in the limitations [lines 503-506].*

Lines 446 – you could also mention the very variable regional estimates of brucellosis prevalence presented in this reference.

*We have edited the sentence accordingly [lines 494-495].*

Lines 447 – See earlier point on the distinction between febrile individuals and individuals tested for brucellosis. Data on these proportions and the factors that determine testing practices (which are likely to be influenced by test performance) would be valuable in performing a next step analysis of cost-effectiveness also.

*We have tried to clarify that the study results refer to febrile patients who would be tested for brucellosis based on clinical judgement [lines 495-496]. We also acknowledge that testing practices might be influenced by the test performance, and that this merits future research [lines 486-491].*

Line 455 – the focus on neurobrucellosis is a little strange here. What proportion of brucellosis presentations have neurological signs in this context? How relevant is this focus as compared to say joint manifestations? How many facilities are equipped for safe testing of CSF?

*We have removed the section on neurobrucellosis.*

Line 477 – given the existence of abundant existing data on the superior performance of the RBT vs FBAT tests what other factors do you think might be important in achieving a change in testing behaviour? Is ‘realisation’ of the poor performance of FBATs the only factor?

*We think that another important factor is the government’s endorsement, and have included a statement on this in the concluding paragraph [lines 521-523].*

**SUMMARY & GENERAL COMMENTS**

**Reviewer #1:** The objective of the work submitted is to assess the comparative performance and cost-effectiveness of the Rose Bengal (RBT) and Febrile Antigen Brucella Agglutination (FBAT) tests for diagnosing human brucellosis to support hospitals in Kenya in creating evidence regarding the most appropriate diagnostic strategy. Overall, the study is well focused and the manuscript well written. The study highlights the misdiagnosing problems generated by FBAT and points out the RBT as mainstay diagnostic test for human brucellosis. Moreover, authors also identify the need for harmonization of treatment guidelines in Kenyan hospitals. Conclusions achieved add further evidence to the usefulness of RBT, an often neglected and misunderstood test, particularly in brucellosis endemic areas of resource limited countries. Hence, this work is worth to be published to support hospital decision makers in resource limited settings to implement appropriate diagnostic methods and treatments.

*Thank you.*

However, several gaps and important inconsistencies have been identified (see methods and results sections), and therefore, to be acceptable for publication the manuscript should be submitted to a careful review. To this end, my comments are made in the hope that they will be well-taken and useful to the authors.

*We found the comments very useful, and hope that we have addressed them suitably.*

**Reviewer #2:** Regarding the diagnosis of brucellosis in Kenya, the authors compared the diagnostic performance and cost-effectiveness of the routinely used FBAT with that of the Rose Bengal Test (RBT) and provided the evidence that the RBT was a more cost-effective diagnostic test. RBT has been widely adopted in many countries, from the economic perspective the authors conducted the cost-effectiveness in detail to indicate the superiority of RBT, while there must be several factors that affect the application of RBT in Kenya. The authors should add some discussion about these factors, and only by addressing these factors, the application of RBT can be promoted.

*We have included a statement on the importance of the government’s endorsement in the discussion [lines 464-466] and concluding [lines 521-523] sections.*

**Reviewer #3:** This paper presents useful data and analyses on an important topic with considerable public health and policy relevance. The study has some limitations but makes a useful contribution to the literature.

*Thank you.*

Some modifications are needed. Key points that apply throughout are the need to clarify that:

1) These data come from multiple different FBATs not one single test and

*We have included information on the different FBAT kits used in the participating hospitals in Table 2, and have discussed this in the results [lines 309-322] and discussion [lines 413-416] sections.*

2) The problems with the assumptions in the cost calculations that all FBAT positive individuals would be put on the recommended treatment for brucellosis. I can see the need to make these assumptions but if more precision in the estimation is not possible (see some suggestions in comments), then these limitations do at least need to be more explicitly recognised.

*We have acknowledged the limitations of these assumptions in the methods [lines 238-242] and discussion [lines 279-291] section.*

The balance of content across sections also needs review. There is much more content in the results and discussion sections on antimicrobial treatment regimens than indicated based on the introduction and methods and some of the results presented have limited detail and set up in the methods sections.

*We have revised the text to include more information on antimicrobial treatment regimens in the introduction [lines 106-111] and methods [lines 149-152] sections. We have also re-organized the text to improve the alignment between objectives, methods, and results.*

**Reviewer #4:** I appreciate the work of Alumasa et al and in my opinion this work presents several important information that can be useful in countries where Brucellosis is endemic. According to the authors, the FBAT test is widely used in many countries in Africa including Kenya for the diagnosis of Brucellosis even though it is unspecific and costly, therefore has resulted in over diagnosing and unnecessary treatments in hospitals. The work attempted to determine the type and cost of treatment for Brucellosis through the use of mathematical modelling to simulate cost outcomes based on the various presented scenarios.

*Thank you.*

However, I am concerned about the vagueness of the method of determining the diagnostic accuracy of FBAT. I have included some suggestions for the authors to follow through.

*We acknowledge that there was vagueness in how we presented the diagnostic accuracy of the FBAT. We have revised the text to clarify that the diagnostic parameters used in the cost-effectiveness analysis are based on previously published literature [lines 190-198]. We have also removed the section describing the relative sensitivity and specificity of the FBAT based on our study data to avoid confusion.*

I am also concern about the length and wordiness of the manuscript. In my opinion the paper can be made significantly more concise and less verbose for the convenience of the reader.

*We have removed a number of paragraphs, particularly in the introduction and discussion sections. We have also removed one Table (what was previously Table 3).*

I recommend professional editing to improve the flow and readability of the manuscript.

*We have revised the text to improve the flow and readability of the manuscript.*

Please improve the title of this manuscript as it is not reflecting the study scope and content.

*The manuscript title has been revised accordingly [lines 1-2].*
